# Support of BCP-ALL-cells by autologous bone marrow Th-cells involves induction of AID expression but not widespread AID off-target mutagenesis

**DOI:** 10.1007/s00262-020-02835-x

**Published:** 2021-01-28

**Authors:** Sabrina Traxel, Julia Lehmann, Stefanie Richard, Semjon Sidorov, Felix Niggli, Christoph Berger, David Nadal, Simone Bürgler

**Affiliations:** Experimental Infectious Diseases and Cancer Research, Children’s Research Center, University Children’s Hospital Zurich, Zurich, Switzerland

**Keywords:** B-cell precursor acute lymphoblastic leukemia, T-helper cells, Activation-induced cytidine deaminase, Mutational signature

## Abstract

B-cell precursor acute lymphoblastic leukemia (BCP-ALL) is the most common childhood malignancy. The two-step BCP-ALL pathogenesis requires in utero-induced chromosomal aberrations and additional mutagenic events for overt leukemia. In mouse models, activation-induced cytidine deaminase (AID/*AICDA*) was suggested to contribute to BCP-ALL pathogenesis by off-target mutagenic activity. The role of AID in patients, however, remains unclear. Moreover, AID is usually not expressed in precursor B-cells but in germinal center B-cells, where it is induced upon T-helper (Th) cell stimulation. We have previously demonstrated that autologous Th-cells supportively interacted with BCP-ALL-cells. Here, we hypothesize that this interaction additionally induces AID expression in BCP-ALL-cells, leading to off-target mutagenic activity. We show that co-culture with autologous bone marrow Th-cells induced high *AICDA* expression in primary BCP-ALL-cells. This induction was mediated by a mechanism similar to the induction in mature B-cells involving IL-13/Stat6, CD40L/NF-κB and TGFβ/Smad2/3 signaling. Even though Th-cell-induced AID seemed to be active in vitro in a BCP-ALL reporter cell line, extensive mutational signature analysis revealed no major contribution of AID activity to the mutational landscape in BCP-ALL patients. AID activity was neither detected in mutation clusters nor in known AID targets. Moreover, no recurrently mutated gene showed a relevant enrichment of mutations in the AID motif. Together, the lack of AID-induced mutational consequences argues towards a Th-cell-promoted yet AID-independent BCP-ALL pathogenesis and favors therapeutic research focusing on Th-cell-derived support of BCP-ALL-cells rather than AID-induced effects.

**Supplementary Information:**

The online version contains supplementary material available at 10.1007/s00262-020-02835-x.

## Introduction

B-cell precursor acute lymphoblastic leukemia (BCP-ALL), the most common pediatric malignancy [1], arises from the expansion of precursor B-cells in the bone marrow (BM). Its pathogenesis is thought to be a two-step process [2]. The first step is characterized by chromosomal aberrations with prenatal origin, such as ETV6-RUNX1 [3]. This translocation is present in 1–5% of newborns but only 1% thereof develop overt leukemia [4], i.e., after the acquisition of secondary mutations. Both epidemiological and recent mechanistic studies suggest that a delayed exposure to common infections is involved in the second step [2, 5, 6]. At a later stage of BCP-ALL, additional mutagenesis may induce mutations associated with therapy resistance [7]. The precise mechanisms leading to secondary and therapy resistance-associated mutations are unclear.

The enzyme activation-induced cytidine deaminase (AID encoded by *AICDA*), usually expressed in germinal center (GC) B-cells, is essential for beneficial mutations in the Ig loci during somatic hypermutation (SHM) and class switch recombination (CSR). Its expression is induced upon contact with T-helper (Th) cells. In mature B-cells, AID also causes off-target mutations in proto-oncogenes, thereby contributing to both initiation and progression of B-cell malignancies like chronic lymphocytic leukemia (CLL), Burkitt lymphoma and multiple myeloma (MM) [8–11]. In fact, AID expression is associated with worse outcome in a subset of CLL patients [9]. Although scarcely expressed in healthy precursor B-cells, AID accelerates overt leukemia induction in mouse models of BCR-ABL1 and ETV6-RUNX1 BCP-ALL [12, 13]. Moreover, AID expression is associated with higher mutation load in patients with BCR-ABL1 BCP-ALL [14]. Interestingly, AID is not required for leukemia development in the Pax5^+/−^ mouse model [15]. Thus, the presence of AID-induced mutations in patients with BCP-ALL subtypes other than BCR-ABL1 and the mechanism of aberrant AID expression remains unclear.

Following infections, memory Th-cells home to the BM [16]. We have previously shown that these BM Th-cells promote activation and proliferation of BCP-ALL-cells, thereby providing a mechanistic link between infections and leukemia development [17]. Here, we hypothesized that Th-cells induce AID expression, which may lead to off-target mutagenesis in BCP-ALL-cells and contribute to secondary and therapy resistance-associated mutations. Thus, we investigated the potential of autologous Th-cells to induce AID expression in BCP-ALL-cells upon co-culture and the presence of AID-induced mutations in different BCP-ALL subtypes.

## Materials and methods

### Patient materials

The study was performed in accordance with the 1964 declaration of Helsinki and approved by the regional ethics committee (Kantonale Ethikkommision Zürich, KEK-ZH Nr. StV40/05 and Nr. 2014-0701). BM mononuclear cells (BMMCs) were obtained from BM aspirates of pediatric BCP-ALL patients at the University Children’s Hospital Zurich and from the Biobank of the Swiss Paediatric Oncology Group (SPOG) after informed consent. ALL671 BMMCs were derived from an NSG xenograft and a gift from the group of Jean-Pierre Bourquin and Beat Bornhauser (University Children’s Hospital Zurich). Palatine tonsils were derived from healthy children after routine tonsillectomy at the University Children’s Hospital Zurich after informed consent.

### Cell culture

Lymphoblastoid cell lines (LCLs) were generated from tonsillar mononuclear cells as described [18]. Th-cells were isolated from BMMCs and expanded as described [17]. SD1 (gift from Beat Schäfer, University Children’s Hospital Zurich), RS4;11 (DSMZ), Nalm6 (gift from Jean-Pierre Bourquin, University Children’s Hospital Zurich) cells and LCLs were cultured in RPMI-1640 (Sigma Aldrich) supplemented with 10% heat-inactivated fetal bovine serum (hi-FBS, Sigma Aldrich), GlutaMAX™-I, 100 U/ml Penicillin, 100 μg/ml Streptomycin, (all gibco), hereafter called c-RPMI. TOM1 (DSMZ) cells were cultured in cRPMI supplemented with additional 10% hi-FBS. HEK293-T (gift from Beat Schäfer, University Children’s Hospital Zurich) cells were cultured in Dulbecco’s Modified Eagle’s Medium–high glucose (Sigma Aldrich) supplemented with 10% hi-FBS, GlutaMAX™-I, and Penicillin–Streptomycin. All cells were cultured at 37 °C in 5% CO_2_.

### Co-cultures

Rested Th-cells (> 95% purity by flow cytometry) were co-cultured with CD4neg BMMCs at a 1:1 ratio in c-RPMI with 20 U/ml human recombinant IL-2 (Roche Diagnostics) in presence or absence of Dynabeads™ Human T-Activator CD3/CD28 (gibco). After co-culture, leukemia cells were separated with CD19 microbeads and two consecutive columns (Miltenyi Biotec). CD19^+^ isolation efficiency was pre-validated using tonsillar B-cell/Th-cell co-cultures and was > 80%.

### Th-cell derived stimuli

BCP-ALL-cells were stimulated with recombinant human IL-13 (100 ng/ml), recombinant human TGF-β1 (10 ng/ml), and recombinant IFN-γ (50 ng/ml, all Peprotech). Stimulation with HA-tagged recombinant human CD40 Ligand (CD40L) (500 ng/ml) was complemented with anti-HA antibody (200 ng/ml, both R&D Systems) enabling multimer formation.

### siRNA knock-down

RS4;11 cells were electroporated using the NEON™ Transfection System (Thermo Fisher Scientific) with Silencer™ Select siRNAs (Thermo Fisher Scientific) targeting *RELA* (s11916), *RELB* (s11918), *SMAD3* (s8401), *STAT6* (s13542) and *SMAD2* (1:1 ratio of s8397 and s8398). A non-targeting siRNA (4390843) was used as a negative control.

### qRT-PCR

RNA from primary samples was isolated using Quick-RNA Microprep kit (Zymo Research). RNA from cell lines was isolated using RNeasy Miniprep kit (Qiagen) with subsequent DNA-free™ Kit treatment (Thermo Fisher Scientific) or Quick-RNA Microprep kit. First strand cDNA was generated using High-Capacity cDNA Reverse Transcription Kit (Thermo Fisher Scientific). qRT-PCR was performed using TaqMan™ Gene Expression Master Mix (Thermo Fisher Scientific) on a CFX384 qPCR system (Bio-Rad Laboratories). Gene expression was measured using pre-designed assays from Thermo Fisher Scientific for *AICDA* (Hs00757808_m1), *UNG* (Hs01037093_m1), *MSH2* (Hs00954125_m1), *MSH6* (Hs00943000_m1), *REV1* (Hs00249411_m1), *APEX1* (Hs00172396_m1), and *POLH* (Hs00197814_m1) and from Integrated DNA Technologies for *POLB* (Hs.PT.58.14431563), *BCL6* (Hs.PT.56a.19673829.g), and *APEX2* (Hs.PT.58.46813622). For primary cells, gene expression was normalized to the geometric mean of *HMBS* (Hs00609297_m1, Thermo Fisher Scientific), *GUSB* (Hs.PT.58v.27737538), *TBP* (Hs.PT.39a.22214825) and *YWHAZ* (Hs.PT.39a.22214858, all Integrated DNA Technologies) using the dCT method. For cell lines, *AICDA* expression was normalized to the geometric mean of *HMBS* and *GUSB* using the ddCT method. The stability of endogenous control combinations was pre-validated using the R package NormqPCR_1.30.0 [19]. For early experiments, gene expression was normalized to *HMBS* only, and qRT-PCR was performed on a 7900HT qPCR system (see Figure legends, Thermo Fisher Scientific).

#### Pre-amplification

Due to the low RNA yield of primary samples, targets were pre-amplified using TaqMan™ PreAmp Master Mix (Thermo Fisher Scientific). Unbiased target gene pre-amplification was pre-validated. Pre-amplification of genes detected at CT ≥ 35 before pre-amplification was unreliable. Therefore, limit of detection (LOD) was set to LOD_Experiment_ = 35–cyclegain_Experiment_. Experiment specific cycle gain was determined with a sample analyzed before and after pre-amplification. CTs ≥ LOD were replaced with LOD_Experiment_.

### ELISA

Th-cell IL-13 secretion was measured after CD3/CD28 stimulation in cRPMI with 20 U/ml IL-2 using the Human IL-13 Uncoated ELISA kit (Invitrogen). TGFβ secretion was measured after CD3/CD28 stimulation in serum-free AIM V® medium (gibco) with 20 U/ml IL-2 using the Human/Mouse TGF beta 1 Uncoated ELISA kit (Invitrogen).

### Flow cytometry

Th-cells were stained with Zombie Red viability dye, anti-CD19-BV421 (HIB-19), anti-CD4-APC-FIRE (RPA-T4) (all Biolegend^®^) and anti-CD40L-APC (24–31, eBioscience™) or appropriate isotype control. Cells were fixed using BD CytoFix/CytoPerm™ (BD Biosciences) and acquired on BD LSR Fortessa (BD Biosciences).

Tonsillar naïve and GC B-cells were sorted as follows: Tonsillar B-cells were isolated as described [18]. Naïve B-cells (CD3^−^ CD19^+^ CD38^low^ IgD^+^) and GC B-cells (CD3^−^ CD19^+^ CD38^high^ IgD^−^) were sorted using BD FACSAria™ Fusion (BD Bioscience) after staining with Zombie Red viability dye, anti-CD3-FITC (UCHT-1), anti-CD19-BV421 (HIB-19), anti-IgD-APC (IA6-2, all Biolegend) and anti-CD38-PE (HIT2, BD Biosciences). The purity after the sort was at least 84%.

### Western blotting

For total protein extraction, cells were lysed in urea lysis buffer [8 M Urea, 0.5% Triton-X, cOmplete Tablet Mini, PhosphoStop (both Roche Diagnostics)]. Cellular fractionation lysates were prepared as described [20], complemented with longer centrifugation and washing of the nuclear pellet for higher purity. Proteins were separated on 4–12% NuPage Gels (Thermo Fisher Scientific) and transferred to nitrocellulose membranes (GE Healthcare Life Science). Primary antibodies against AID (#4949), β-actin (#4967), p65 (#4764), p100/p52 (#4882), RelB (#4922), αTubulin (#3873, all Cell Signaling Technologies), Smad2/3 (GTX111123), Stat6 (GTX113273, both GeneTex) and Phospho-Stat6 (686002, Biolegend^®^) were used. Primary antibodies were detected using HRP-labeled secondary antibodies (Cell Signaling Technologies) and Amersham ECL™ Western Blotting reagent (GE Healthcare Life Science) or Supersignal™ West Femto Maximum Sensitivity Substrate (Thermo Fisher Scientific) using a ChemiDoc Imaging System (Bio-Rad Laboratories).

### Microarray analysis

Microarray data were published before [21] (accession GSE13576). *AICDA* gene expression in patients without relapse, with relapse before 24 months, and relapse after 24 months was analyzed.

### St. Jude patient samples

To analyze the presence of AID mutational signature, whole-genome sequencing (WGS) data generated by the St. Jude Children’s Research Hospital—Washington University Pediatric Cancer Genome Project were used [22]. See Table 1 for clinical patient data.

**Table 1:** Clinical data of St. Jude patients

	*n*	%
	243	100
Chromosomal aberrations		
E2A-PBX1	21	8.6
ERG	25	10.3
ETV6-RUNX1	49	20.2
Hyperdiploid	53	21.8
Hypodiploid	24	9.9
BCR-ABL1	37	15.2
others	34	14.0
Age		
Median	6.55	
Range	1.02–19.68	
Not determined	20	8.2
Sex		
Female	101	41.6
Male	138	56.8
Not determined	4	1.6

#### Mutational signatures

Single-nucleotide variants (SNVs) from WGS data were called by the St. Jude in-house somatic variant analysis pipeline. Mutational signature analysis was performed by fitting the signatures SBS1-30 [23], SBS84, and SBS85 (Cosmic version 3 [24]) using the R package MutationalPatterns 1.10.0 [23]. Refitting quality was assessed using cosine similarity by comparing the initial with the reconstructed mutation profile. AID-target genes were defined as human orthologs of mouse AID off-target genes [25].

#### Alternative AID-target genes

Non-synonymous mutations were identified using the R package VariantAnnotation 1.30.1 [26] and analyzed for their 3 bp context. Mutation frequency within and outside the AID-target motif (RC > NY) was compared to the frequency expected by random mutation distribution (motif frequency in the gene sequence). *KRAS* mutations of other cancer types were collected from the COSMIC database [27].

### RNA sequencing analysis

For 184 of the patients analyzed for AID activity, RNA sequencing data were published before [28] and can be accessed at https://viz.stjude.cloud/StJude/visualization/pax5-driven-subtypes-of-b-progenitor-acute-lymphoblastic-leukemia-heatmap. *AICDA* gene expression was analyzed in patients with and without BCR-ABL1 translocation.

### In vitro AID activity

#### Reporter construct

To generate the reporter and control constructs [pL40C_PGKintron_EGFP(Stop)_BSD], constructs previously described [pCru5-EGFP(Stop)-IRES-Puro, gift from Matthias Wabl, University of California] [29], were cloned into the lentiviral vector pL40C_PGKintron_Cas9_Green (Addgene #134966) and puromycin resistance was replaced by a blasticidin resistance from pLX-sgRNA (Addgene #50662).

#### Reporter cell line generation

Lentivirus was produced using calcium phosphate transfection of HEK293T cells with pL40C_PGKintron_EGFP(Stop)_BSD, pVSV-G (Clontech Laboratories) and psPax2 (Addgene #12260). RS4;11 EGFP-Stop and EGFP were generated by spinoculation with lentiviral supernatant and 5 μg/ml polybrene (Merck Millipore). Transduced cells were selected in 10 μg/ml blasticidin (InvivoGen) for 6 days and further maintained at 5 μg/ml blasticidin.

#### Reporter assay

Cells were harvested every fourth day and stained with 7-AAD (Biolegend^®^) for flow cytometric analysis using BD LSR Fortessa.

#### Sanger sequencing

To validate Stop codon reversion, EGFP high, low and negative cells were sorted using BD FACSAria™ Fusion (BD Bioscience) and outgrown. Genomic DNA was isolated using QIAmp^®^ DNA Blood Mini Kit (Qiagen). EGFP was amplified using nested PCR with Phusion High-Fidelity DNA Polymerase and ligated into pCR^®^ 2.1 vector (The Original TA Cloning Kit, both Thermo Fisher Scientific). Five clones per population were analyzed by Sanger sequencing (Microsynth).

### Statistical analysis

Statistical analysis was performed using GraphPad Prism 8.0.3 (GraphPad Software, LLC) or R 3.6.1, as indicated in the figure legends.

## Results

### BM Th-cells induce *AICDA* expression in BCP-ALL-cells

**Fig. 1:**
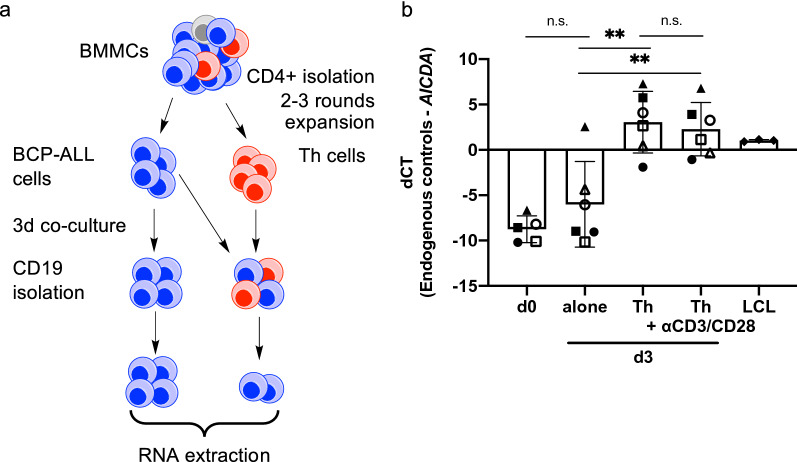
Th-cell co-culture induces *AICDA* expression in BCP-ALL-cells. **a** Scheme of co-culture experiment. **b** Primary BCP-ALL-cells were cultured either alone or in co-culture with expanded autologous Th-cells. Th-cells used for co-culture were either rested or stimulated using anti-CD3/CD28 beads. *AICDA* expression in CD19^+^ cells was determined using qRT-PCR after preamplification of target genes. Shown are mean ± SD of dCT values normalized to the geometric mean of *HMBS*, *TBP*, *GUSB* and *YWAHZ*. LCLs were used as a positive control. Different symbols represent different patients. *p* values were calculated using one-way ANOVA with Tukey correction for multiple comparison. *p* > 0.05 not significant (n.s.), *p* < 0.01**

We previously showed that autologous BM Th-cells activate and support proliferation of BCP-ALL-cells [17]. Here, we asked whether such interactions lead to *AICDA* expression in BCP-ALL-cells. Thus, we separated BMMCs into BCP-ALL-cells and Th-cells, cultured purified BCP-ALL-cells alone or with expanded Th-cells, and assessed *AICDA* expression in subsequently purified BCP-ALL-cells by qRT-PCR (Fig. 1a). Whereas BCP-ALL-cells cultured alone showed *AICDA* expression close to or below detection limit, their co-culture with autologous BM Th-cells resulted in significantly higher *AICDA* expression (Fig. 1b). Thus, analogous to their interaction with mature B-cells, Th-cells are, in fact, able to induce *AICDA* expression in BCP-ALL-cells that derive from B-cell precursors. This expression was independent of exogenous Th-cell activation by CD3/CD28 stimulation, confirming our previous findings that BCP-ALL-cells themselves exhibit the potential to activate Th-cells.

### IL-13, TGFβ and CD40L mediate Th-cell-induced *AICDA* expression in BCP-ALL-cells

Next, we aimed to identify mediators of Th-cell-induced *AICDA* expression. IL-4, TGFβ, and CD40L are established Th-cell-derived inducers of *AICDA* expression in mature B-cells [30]. Our previous cytokine profiling of BM Th-cells from BCP-ALL patients revealed high IFNγ secretion, but importantly also IL-13 secretion and *TGFB1* gene expression [17]. While IFNγ has not been involved in *AICDA* expression, IL-13 may induce *AICDA* expression similarly to IL-4 [31]. Thus, we performed ELISA to confirm high secretion of IL-13 by BM Th-cells (Supplementary Fig. 1a). TGFβ was secreted at low concentration, likely due to decreased Th-cell viability in serum-free culture and thus can be expected to be higher in vivo (Supplementary Fig. 1b). Moreover, CD40L surface expression in activated Th-cells was reduced upon co-culture with BCP-ALL-cells, indicating contact-induced downregulation of CD40L (Supplementary Fig. 1c). Collectively, we showed that BM Th-cells from BCP-ALL patients express and secrete *AICDA*-inducing stimuli.

**Fig. 2:**
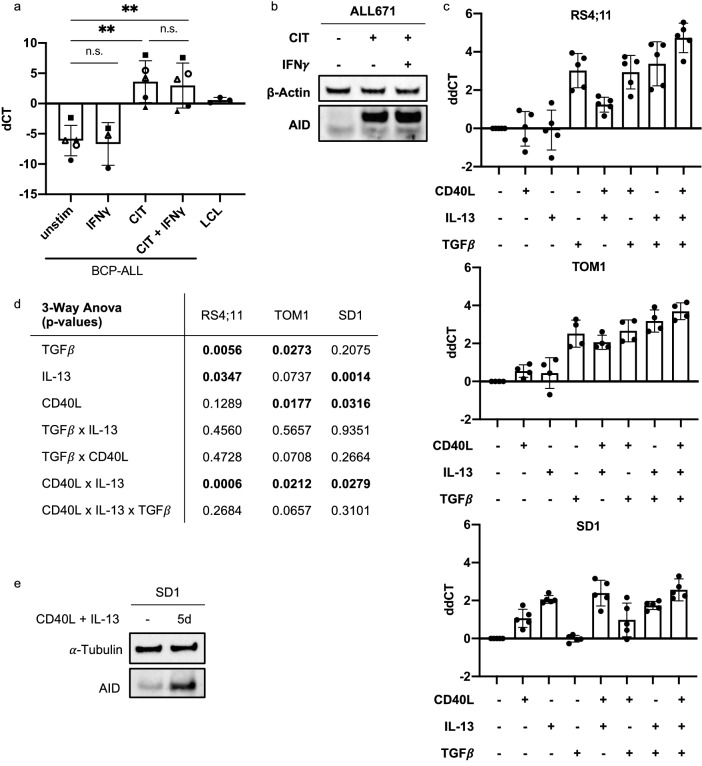
IL-13, TGFβ and CD40L mediate Th-cell-induced *AICDA* expression in BCP-ALL-cells. **a** Primary BCP-ALL-cells were stimulated with CIT ± IFNγ for 3d. *AICDA* expression was determined using qRT-PCR after preamplification of target genes. Shown are mean ± SD of dCT values normalized to the geometric mean of *HMBS*, *TBP*, *GUSB* and *YWAHZ*. Different symbols represent different patients. *p *values were calculated using a mixed-effect analysis with Sidak correction for multiple comparison. *p* > 0.05 not significant (n.s.), *p* < 0.01** **b** Xenograft ALL671 BMMCs were stimulated using CIT ± IFNγ for 5d. Total AID protein expression was assessed using western blotting. β-actin was used as loading control. Western blot of primary cells was performed once. **c** RS4;11, TOM1 and SD1 cells were stimulated for 3d using different combinations of CD40L, IL-13 and TGFβ. *AICDA* expression was determined using qRT-PCR. Shown are mean ± SD of ddCT values normalized to the geometric mean of *HMBS* and *GUSB* and to unstimulated cells. Dots represent different replicates. **d** *p *values in the table were calculated from dCT values using three-way ANOVA with Geisser-Greenhouse correction for sphericity. **e** SD1 cells were stimulated using CD40L and IL-13 for 5d. AID protein expression was analyzed using western blotting. α-tubulin was used as loading control. Shown is a representative result of five replicates

Then, to test whether these stimuli induce *AICDA* expression in BCP-ALL-cells, we stimulated primary BCP-ALL-cells with a combination of CD40L, IL-13, and TGFβ (hereafter abbreviated CIT). Similar to Th-cell co-culture, CIT stimulation induced high *AICDA* mRNA and protein expression (Fig. 2a, b). As expected, IFNγ neither induced *AICDA* expression alone nor further increased CIT induced *AICDA* expression. To characterize their individual impacts on *AICDA* expression, we next stimulated four BCP-ALL cell lines (RS4;11, TOM1, SD1, and Nalm6) with combinations of CD40L, IL-13, and TGFβ (Fig. 2c, d). In these cell lines, which we used due to the limited cell numbers in patient samples, *AICDA* induction was lower than in primary BCP-ALL-cells. In both RS4;11 and TOM1 cells, TGFβ, and IL-13/CD40L combination had a significant effect on *AICDA* expression, while IL-13 in RS4;11 and CD40L in TOM1 had a significant although only moderate effect. In SD1 cells, IL-13 and CD40L alone significantly increased *AICDA* expression and had a synergistic effect. Conversely, TGFβ alone or in combination with other stimuli did not induce *AICDA* expression in SD1 cells (Fig. 2c, d). IL-13/CD40L combination also increased AID protein expression in SD1 cells (Fig. 2e), while AID protein expression could not be detected in TOM1 and RS4;11 cells (not shown). Noteworthy, none of the stimuli induced *AICDA* expression in the cell line Nalm6, neither on mRNA nor on protein level (not shown). Taken together, we have identified Th-cell-derived IL-13, TGFβ, and CD40L as important mediators of Th-cell-induced *AICDA* expression in BCP-ALL-cells. Nevertheless, the combination of stimuli required for *AICDA* induction and the extend of induction varied between cell lines and patient samples, suggesting that different BCP-ALL subtypes may differ in their response to *AICDA*-inducing stimuli, or may not upregulate *AICDA* at all. Due to the small number of primary samples and the fact that the cell lines used here do not cover all the molecular subtypes of pediatric BCP-ALL (such as the most common subtypes *ETV6-RUNX1* and hyperdiploidy), further studies will be needed to assess the impact of the molecular subtype on the extend of *AICDA* upregulation.

### Th-cell-induced *AICDA* is transcriptionally regulated via canonical NF-κB, Stat6 and Smad2/3 signaling in BCP-ALL-cells

**Fig. 3:**
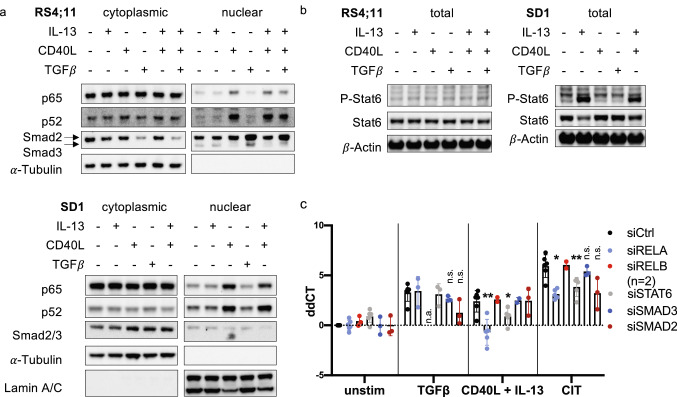
Th-cell-induced *AICDA* is transcriptionally regulated via canonical NF-κB, Stat6 and Smad2/3 signaling in BCP-ALL-cells. **a**, **b** RS4;11 and SD1 cell lines were stimulated for 2 h with CD40L, IL-13 and TGFβ. **a** Localization of transcription factors in cytoplasmic and nuclear fraction was analyzed using western blotting. α-Tubulin and Lamin A/C were used as a loading and fractionation controls. RS4;11 cells do not express Lamin A/C. **b** Phosphorylation of Stat6 was analyzed using western blotting. β-actin was used as a loading control. **c** Transcription factors were knocked-down in RS4;11 cells by electroporation of siRNA for 48 h and stimulated with CD40L, IL-13 or TGFβ for 24 h. *AICDA* expression was analyzed using qRT-PCR. Shown are ddCT normalized to *HMBS* and siCtrl/unstimulated condition. *p *values were calculated using a mixed-effect analysis by comparing siCtrl and with targeting siRNA with same stimulation. Sidak correction for multiple comparison was used. *p* > 0.05 not significant (n.s.), *p* < 0.05*, *p* < 0.01**, *n.a. *not analyzed

Th-cell-induced expression of *AICDA* in precursor B-cells or BCP-ALL-cells has not been described so far. Thus, we aimed to characterize the mechanism(s) of *AICDA* expression in BCP-ALL-cells to gain further insight into BCP-ALL pathogenesis. Extensive analysis of the transcriptional regulation of *AICDA* in a murine mature B-cell lymphoma cell line had revealed that CD40L, IL-4, and TGFβ induce *AICDA* expression via NF-κB, Stat6 and Smad3/4 signaling, respectively [32]. Therefore, we analyzed the activation of above-mentioned transcription factors in two BCP-ALL cell lines (SD1 and RS4;11) upon stimulation with different combinations of CD40L, IL-13, and TGFβ. As expected, CD40L stimulation activated both canonical (p65 nuclear localization) and non-canonical (p52 nuclear localization) NF-κB pathways in both cell lines (Fig. 3a). TGFβ activated Smad3 in RS4;11 but not in SD1 cells, consistent with the lack of TGFβ-induced *AICDA* upregulation in the latter. IL-13 activated Stat6 in SD1 cells, as we had hypothesized based on the fact that IL-13 and IL-4 share signaling pathways [33] (Fig. 3b). Unexpectedly, no Stat6 activation was detectable in RS4;11 cells. Next, we functionally assessed the importance of Smad3, Stat6, and NF-κB in RS4;11 cells by siRNA knock-down and subsequent stimulation. Knock-down efficiency was validated by western blotting (Supplementary Fig. 2). Even though we did not detect Stat6 phosphorylation, the combined effect of CD40L and IL-13 on *AICDA* expression seemed to be mediated by Stat6 (Fig. 3c). Both Stat6 and canonical NF-κB knock-down (siRelA) almost completely abrogated combined CD40L/IL-13-induced *AICDA* expression, indicating a cross-talk of these pathways. The precise role of non-canonical NF-κB (siRelB) remained unclear due to incomplete knock-down (Supplementary Fig. 2). TGFβ-induced *AICDA* expression was only minimally reduced by Smad3 knock-down. Instead, TGFβ also activated Smad2 (Fig. 3a), and Smad2 knock-down tended to reduce TGFβ-induced *AICDA* expression (Fig. 3c). In conclusion, we suggest that transcriptional regulation of *AICDA* in BCP-ALL-cells closely resembles its regulation in murine mature B-cells.

### AID-induced mutations are not commonly found in BCP-ALL patient samples

**Fig. 4:**
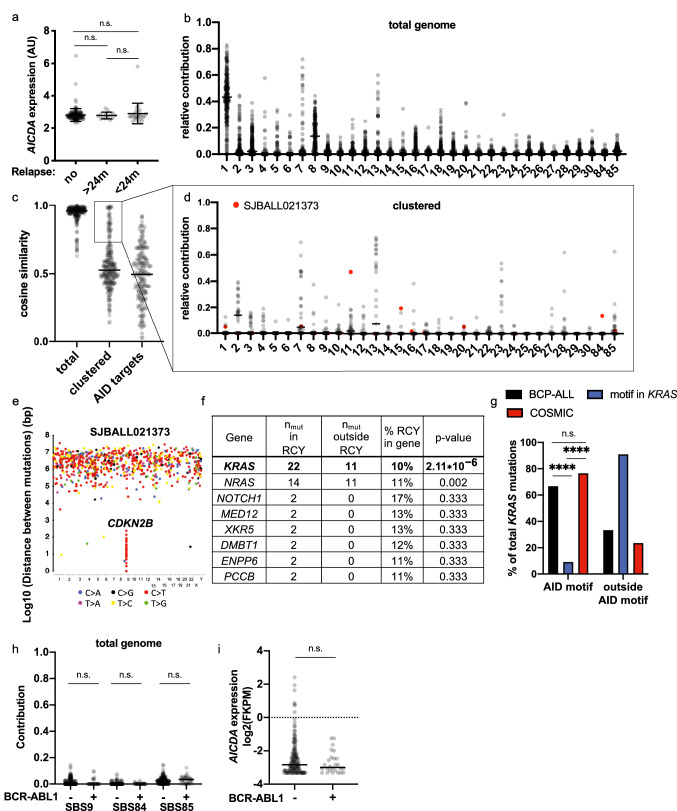
AID-induced mutations are not commonly found in BCP-ALL patients samples. **a** *AICDA* expression was analyzed by microarray in xenografts generated from diagnostic samples of patients with no, relapse after 24 months and relapse before 24 months. *AICDA* expression for each patient and mean ± SD are shown. *p *values were calculated using one-way ANOVA with Tukey correction for multiple comparison. *p* > 0.05 not significant (n.s.), **b**–**d** Values of each patient and median values are shown. **b** Relative contribution of mutational signatures to mutations in total genome was assessed using R package MutationalPatterns in WGS data. **c** Cosine similarity of original mutation profile compared to the reconstructed mutation profile. **d** Relative contribution of mutational signatures to clustered mutations is shown for patients with cosine similarity > 0.75. Relative contribution in patient SJBALL021373 is depicted in red. **e** Rainfallplot of mutations in patient SJBALL021373 was generated using R package karyoploteR. **f** Table of genes with most mutations in AID motif (RC > NY). *p* values were calculated using Fisher’s exact test by comparing observed mutations (n_mut_ in RCY) to expected AID motif mutations for each gene according to the frequency of AID motif in each gene (% RCY in gene). Genes with statistically significant enrichment of AID motif mutations after Benjamini–Hochberg correction are depicted in bold. **g** Frequency of AID motif mutations in *KRAS* in BCP-ALL patients, in COSMIC database and according to distribution of motif in *KRAS* sequence. *p *values were calculated using Fisher’s exact tests. *p* > 0.05 not significant (n.s.), *p* < 0.0001**** . **h** Relative contribution of mutational signatures to mutations in total genome was assessed using R package MutationalPatterns in WGS data. *p* values were calculated using Kruskal–Wallis test and Dunn’s test for multiple comparison *p* > 0.05 not significant (n.s.). **i** *AICDA* expression was analyzed by RNA sequencing in pediatric BCP-ALL patients. Values for each patient and median are shown. *p *values were calculated using Mann–Whitney test. *p* > 0.05 not significant (n.s.)

Following demonstration that Th-cells induce AID expression in BCP-ALL-cells, we searched for evidence of AID activity in BCP-ALL patient samples. We hypothesized *AICDA* expression to be associated with higher mutation load and worse outcome. Analysis of an existing microarray dataset [21], however, showed generally low *AICDA* expression, irrespective of the patients’ outcome (Fig. 4a). Nevertheless, *AICDA* might be expressed only in a small fraction of cells at a given time point, and thus escape microarray detection while being sufficient to cause pathogenesis-relevant mutations. Therefore, we asked whether, at diagnosis, AID-induced mutations are detectable in expanded clones of primary BCP-ALL-cells. Thus, we assessed the activity of AID and other mutational processes by mutational signature analysis in WGS data of 243 BCP-ALL patients. Our refitting approach identified similar signature contributions as previously identified by de novo mutational signature extraction [34] (Fig. 4b). Analysis of the contribution of AID-associated signatures 9, 84, and 85, however, suggested that AID does not lead to widespread mutagenesis. Nevertheless, due to its localized action, AID activity is often detected only in (off-)target genes and in regions with clustered mutations [8, 11, 35]. Accordingly, we next assessed mutational signature contribution in clustered mutations, defined as two mutations localized within 1000 bp. Low numbers of clustered mutations (median = 10) led to a low refitting quality. We, therefore, restricted our analysis to patient samples with a cosine similarity > 0.75 (Fig. 4c). In these samples, most clustered mutations were caused by APOBEC3 mutagenesis (Signatures 2 and 13) (Fig. 4d), known to induce clustered mutations [36]. Additionally, we detected signature 7, likely due to its induction of CC > TT mutations. Collectively, we successfully identified signatures that typically induce clustered mutation. Nonetheless, we found canonical AID activity (Signature 84) in patient sample SJBALL02137 (Fig. 4d), where we identified a kataegic region on chromosome 9 comprising 40 C > T mutations in the *CDKN2B* locus. 40% of these mutations were in the RC > NY motif (Fig. 4e). Notably, *CDKN2B* is an AID target and is often inactivated in BCP-ALL patients [14, 37, 38]. Therefore, AID seems to induce relevant mutations only in rare cases.

Next, we analyzed mutations in Ig loci and described AID off-target genes [25] (Fig. 4c). As shown by the cosine similarity, the even lower number of mutations made mutational signature refitting unreliable. Since AID off-target gene mutation depends on high transcription [39], AID may not target the same genes in BCP-ALL-cells that were described to be off-targets in mature B-cells. Thus, we aimed to identify non-synonymous mutations targeted by AID. We found that the AID-target motif (RC > NY) was significantly enriched in *KRAS* mutations (Fig. 4f, g), but the same target motif was enriched in *KRAS* mutations in the COSMIC database, mostly representing mutations in non-B-cell malignancies (Fig. 4g). Moreover, *KRAS* mutations are restricted to a few hotspots [40]. This suggests that functionally relevant base pairs rather than mutational processes determine the context of *KRAS* mutations. Overall, while a rare AID mutational activity cannot be excluded, our analysis showed no detectable contribution of AID activity to the mutational profile in BCP-ALL patient samples.

### AID-induced mutations and *AICDA* expression are not enhanced in BCR-ABL1 BCP-ALL

Previous studies suggest that particularly BCP-ALL patients of the BCR-ABL1 subtype exhibit high *AICDA*/AID expression and activity [13, 14, 37, 41]. Thus, we aimed to assess the relative contribution of AID-associated mutational signatures in BCR-ABL1 versus other subtypes in our cohort and found a similar contribution of AID-associated mutational signatures to total genome mutations in both groups (Fig. 4h). BCR-ABL1 BCP-ALL samples harbored too few clustered mutations (median = 9) for reliable mutational signature analysis, and none of the mutation clusters in BCR-ABL1 BCP-ALL contained multiple WRC > NY hotspot mutation (and neither less restrictive RC > NY nor WRC > N motifs), indicating that mutation clusters arose by different mutational processes (not shown). In contrast to previous studies [37, 41], published RNA sequencing data of 184 of the patients analyzed for AID activity did not reveal an increased *AICDA* expression in BCR-ABL1 BCP-ALL compared to other subtypes (Fig. 4i) [28]. In conclusion, we did not observe an increased AID activity in BCR-ABL1 BCP-ALL patients, which might be explained by a lack of increased *AICDA* expression in our cohort.

### High-fidelity repair and lack of protection from DNA-damage-induced cell death by BCL6 might explain lack of AID activity in BCP-ALL patients

**Fig. 5:**
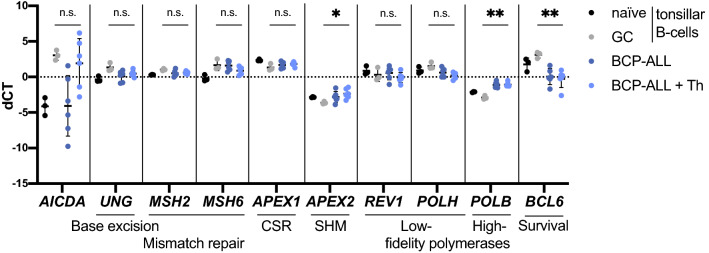
High-fidelity repair and lack of protection from DNA-damage-induced cell death by BCL6 might explain lack of AID activity in BCP-ALL patients. Tonsillar B-cells were sorted into naïve (CD19^+^ CD3^−^ IgD^+^ CD38^−^) and GC (CD19^+^ CD3^−^ IgD^−^ CD38^+^) B-cells. Primary BCP-ALL-cells were treated as in Fig. 1b. Gene expression in CD19^+^ cells was determined using qRT-PCR after preamplification of target genes. Shown are mean ± SD of dCT values normalized to the geometric mean of *HMBS*, *TBP*, *GUSB* and *YWAHZ*. *p *values were calculated using Brown-Forsythe and Welch ANOVA test with Dunnett’s T3 test for multiple comparisons by comparing gene expression in GC B-cells and BCP-ALL-cells co-cultured with Th-cells. *p* > 0.05 not significant (n.s.), *p* < 0.05*, *p* < 0.01**

AID-mediated mutagenesis is initiated by AID-induced cytidine deamination and is followed by the recruitment of and processing by repair proteins. Depending on the recruited proteins, deaminated sites may be repaired with low-fidelity, resulting in mutations, or may be correctly repaired by high-fidelity pathways. Accordingly, mature B-cells entering the GC upregulate genes involved in base-excision and mismatch repair, low-fidelity polymerases, and down-regulate high-fidelity polymerases to increase mutation frequency [42]. Expression of these genes by BCP-ALL-cells, however, is not known. Since lack of AID-induced mutations in BCP-ALL patient samples despite high *AICDA* induction by Th-cells in vitro could be due to insufficient expression of repair proteins, we assessed expression of those genes in BCP-ALL-cells co-cultured with Th-cells. BCP-ALL co-cultured with Th-cells expressed most of the analyzed genes at a similar level as GC B-cells (Fig. 5). Interestingly, however, Th-cell-stimulated BCP-ALL cells showed lower polymerase η (*POLH)* (low-fidelity) and significantly higher polymerase β (*POLB*) (high-fidelity) expression compared to GC B-cells, which may explain the lower mutation frequency despite *AICDA* expression. Additionally, B-cell lymphoma 6 protein (*BCL6)* expression was significantly lower in Th-cell-stimulated BCP-ALL-cells than in GC B-cells. Whereas BCL6 is not directly involved in the repair of AID-induced mutagenesis, it increases the survival of cells undergoing mutagenesis [43, 44]. Thus, AID-induced mutations in BCP-ALL-cells may both be repaired at a higher fidelity as well as lead to DNA damage-induced cell death, which possibly explains a low rate of AID-induced mutations despite high *AICDA* expression.

### AID seems to be active in BCP-ALL-cells in vitro

**Fig. 6:**
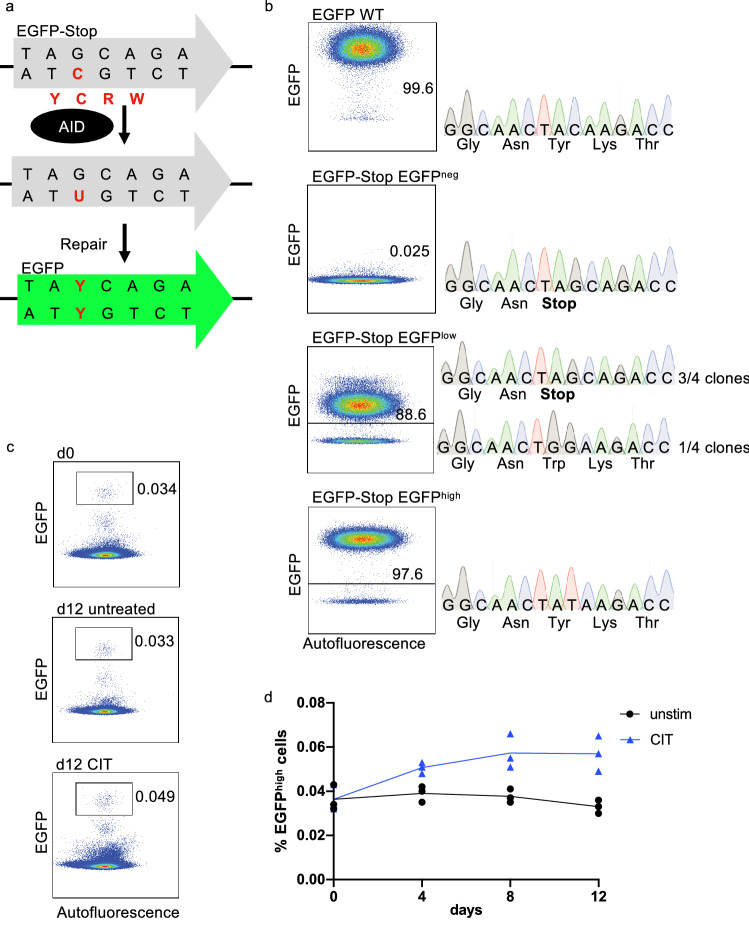
AID seems to be active in BCP-ALL-cells in vitro*.* **a** The lentiviral reporter construct contains an EGFP-sequence with a premature stop codon (EGFP-Stop) within the target motif of AID (WRCY). AID-induced mutagenesis and repair leads to stop codon reversion and to translation of EGFP. **b** RS4;11 cell line was transduced with the EGFP-Stop reporter construct or EGFP control construct. Cells were sorted in EGFP^neg^, EGFP^low^ and EGFP^high^ population and outgrown. Sanger sequencing was performed to assess the reversion of the stop codon. **c** unsorted EGFP-Stop cells were cultured unstimulated or with CIT. Medium was changed every second day. **d** Quantification of EGFP^high^ cells during stimulation

To further investigate the activity of Th-cell-induced AID in BCP-ALL-cells, we generated an RS4;11 reporter cell line, where AID activity leads to the reversion of a premature Stop codon and thereby to EGFP translation (Fig. 6a). After transduction, we observed three populations: one with no, one with low and one with high EGFP expression. Sanger sequencing of the sorted populations showed that only the EGFP^high^ but not the EGFP^low^ population arose by stop codon reversion (Fig. 6b). CIT stimulation of one reporter cell line led to an increase of the EGFP^high^ population, suggesting induction of mutagenic activity (Fig. 6c, d). Nevertheless, no such mutagenic activity could be observed in six newly generated reporter cell lines (not shown). These results imply that, even though Th-cell stimulation of BCP-ALL-cells in vitro can cause mutations, this is likely to be a very rare event—similar to what we observed in patient samples.

## Discussion

Inappropriate immune response to delayed infections has been proposed to trigger development of overt leukemia. We previously showed that Th-cells, which migrate to the BM after infections, promote activation and proliferation of BCP-ALL-cells, suggesting that Th-cells are the link between infection and overt leukemia [17]. Here, we expand our model and show that this supportive interaction includes induction of high *AICDA* expression by a mechanism similar to that in mature B-cells. Furthermore, we found that this expression is not reflected in AID off-target mutations in patient samples, implying that AID is unlikely to be involved in the development of BCP-ALL. Our results add to the understanding of BCP-ALL pathogenesis and the identification of potential therapeutic targets.

Rare *AICDA* expression in healthy precursor B-cells—at least in mice—seems to be Th-cell-independent and rather caused by toll-like receptor signaling [45]. Our demonstration that BM Th-cells are able to induce *AICDA* expression in human (malignant) precursor B-cells in vitro through mediators and mechanisms similar to those in mature B-cells in GC is unprecedented. This may also be relevant for other B-cell malignancies, such as CLL, where subclonal AID expression and activity have been reported in a CLL subset associated with bad outcome [8]. Interestingly, Th-cells interact with CLL-cells, and microarray analysis suggested that this leads to *AICDA* upregulation in CLL-cells ([46] and personal communication).

Our previous [17] and current work strongly suggests that Th-cells support BCP-ALL-cells and induce *AICDA* expression independently of CD3/CD28 stimulation. Thus, (pre)leukemic cells may incite Th-cell-dependent AID-induced mutagenesis by a (neo)antigen-dependent interaction. Whereas most AID-induced off-target mutations may not affect (pre)leukemic cells, some mutations may lead to a survival advantage, clonal expansion and leukemia progression. To elucidate whether BCP-ALL-cells may indeed acquire such AID-mediated survival advantages, we searched for evidence of AID activity in BCP-ALL patients employing three strategies: (1) mutational signature analysis in clustered mutations, (2) analysis of mutations in known AID-target genes, and (3) identification of alternative AID-target genes in the precursor B-cell context. None of the approaches provided evidence for extensive AID activity in BCP-ALL patients. This contrasts previous findings in BCR-ABL1 and ETV6-RUNX1 preleukemic mouse models, where AID accelerates the progression to overt leukemia [12, 13].

In contrast to previous studies that detected higher *AICDA/*AID mRNA, protein expression, and activity in BCR-ABL1 BCP-ALL than in other subtypes [37, 41], we did not detect such an association in our cohort. Interestingly, BCR-ABL1 is more frequent in adult than in pediatric patients, and previous studies mostly analyzed samples from adult patients for *AICDA*/AID expression: AID protein expression was analyzed in adults only and 8/10 samples analyzed for *AICDA* gene expression were adults, though age data is not available for 28 additional patients [37, 41]. Thus, the mutagenic activity of AID in BCR-ABL1 BCP-ALL may be restricted to adult patients by a yet unknown mechanism.

AID was further suggested to accelerate the pathogenesis of ETV6-RUNX1 BCP-ALL upon the upregulation of AID by infectious stimuli. In this model, preleukemic cells were repetitively stimulated with LPS ex vivo [12]. In contrast to above mouse model, more physiological infectious stimuli induce overt leukemia in a Pax5^+/−^ mouse independently of AID expression [15]. Thus, even though we cannot rule out a role for AID in the formation of structural variants, our data using patient samples strongly favors an AID-independent pathogenesis in humans.

Lack of AID activity and low basal AID expression ex vivo despite the high induction by Th-cells in vitro may reflect rare contact between Th-cells and BCP-ALL-cells in vivo or inhibition of *AICDA* upregulation by other cells of the leukemic microenvironment. Further research using bone marrow biopsies or infection-induced BCP-ALL mouse models [5, 6] is required to address whether and how the frequency and duration of contacts between Th-cells and BCP-ALL-cells correlate with AID expression, and whether and how other cells of the leukemic environment affect AID upregulation and BCP-ALL-cell proliferation. In addition, the majority of AID-induced mutations in preleukemic cells may remain undetected as only a minority of mutations may provide a survival advantage and result in clonal expansion. Nevertheless, the lack of widespread AID activity makes it unlikely that AID caused the transforming mutagenic event. Moreover, due to the unavailability of human preleukemic cells, we analyzed the Th-cell-induced expression of *AICDA* in leukemic cells. Thus, it remains to be determined whether Th-cells induce *AICDA* expression also in preleukemic cells, where AID-activity could lead to mutations detectable at diagnosis. As TLR-signaling induces *AICDA* expression both in healthy and preleukemic precursor B-cells [12, 15], *AICDA* expression in preleukemic cells may mainly depend on their ability to activate Th-cells, possibly by (neo)antigen-presentation. Furthermore, additional stimuli may be required to induce mutations in precursor B-cells. Even though we found that BCP-ALL-cells expressed most genes encoding for repair proteins at a similar level as GC B-cells, increased *POLB* expression could lead to a lower mutation frequency [47]. Additionally, BCP-ALL-cells expressed less *BCL6* than GC B-cells. As one of the most important transcription factors in GC B-cells, BCL6 may regulate co-factors required for AID activity that where not analyzed here. Thus, decreased *BCL6* expression in BCP-ALL-cells may hinder AID-induced mutagenesis by increased DNA damage-induced cell death [43, 44] and lack of co-factors. Nevertheless, SHM in Ig loci has been detected in different subsets that typically do not show *BCL6* expression [12, 37, 48], while we did not detect any Ig loci mutations in 50% of our patient samples (not shown). The high genomic complexity and homology of the Ig loci require special approaches for alignment of WGS data. As we analyzed already called single-nucleotide variants, the frequency of Ig loci mutations in our study may have been underestimated. Further investigation will be required to clarify the prevalence of SHM in different BCP-ALL subsets and the impact of *BCL6* and *POLB* expression on AID activity in BCP-ALL.

In conclusion, our study extends the recent evidence of an AID-independent BCP-ALL pathogenesis in mice [15] to human patient samples. Thus, AID expression in BCP-ALL-cells seems to be merely an indicator of a recent supportive interaction by Th-cells that have migrated to the BM upon an infection rather than a driver of clonal evolution. As a consequence, research aiming at the development of novel therapies for BCP-ALL may focus on targeting the Th-cell-mediated support of BCP-ALL-cells rather than AID-induced effects.

### Supplementary Information

Below is the link to the electronic supplementary material.

Supplementary file1 (PDF 88 KB)

Supplementary file2 (PDF 307 KB)

## Data Availability

Somatic variants identified by whole-genome sequencing of leukemia patients can be requested at the St. Jude Cloud Platform.

## Abbreviations

AID/*AICDA*: Activation-induced cytidine deaminase
BCP-ALL: B-cell precursor acute lymphoblastic leukemia
BCL6: B-cell lymphoma 6 protein
BM: Bone marrow
BMMC: Bone marrow mononuclear cells
CLL: Chronic lymphocytic leukemia
CSR: Class switch recombination
c-RPMI: Complete RPMI medium
GC: Germinal center
LCLs: Lymphoblastoid cell lines
MM: Multiple myeloma
POLH: Polymerase η
POLB: Polymerase β
SHM: Somatic hypermutation
SNV: Single-nucleotide variant
Th-cell: T-helper cell
WGS: Whole genome sequencing

## Code availability

Code used in this study is available from ST upon reasonable request.

## References

[1] Pui C-H, Robison LL, Look AT (2008). Acute lymphoblastic leukaemia. Lancet.

[2] Greaves M (2018). A causal mechanism for childhood acute lymphoblastic leukaemia. Nat Rev Cancer.

[3] Hein D, Borkhardt A, Fischer U (2020). Insights into the prenatal origin of childhood acute lymphoblastic leukemia. Cancer Metastasis Rev.

[4] Mori H, Colman SM, Xiao Z (2002). Chromosome translocations and covert leukemic clones are generated during normal fetal development. Proc Natl Acad Sci.

[5] Rodríguez-Hernández G, Hauer J, Martín-Lorenzo A (2017). Infection exposure promotes ETV6-RUNX1 precursor B-cell leukemia via impaired H3K4 demethylases. Cancer Res.

[6] Martín-Lorenzo A, Hauer J, Vicente-Duenas C (2015). Infection exposure is a causal factor in B-cell precursor acute lymphoblastic leukemia as a result of Pax5-inherited susceptibility. Cancer Discov.

[7] Bhojwani D, Pui C-H (2013). Relapsed childhood acute lymphoblastic leukaemia. Lancet Oncol.

[8] Kasar S, Kim J, Improgo R (2015). Whole-genome sequencing reveals activation-induced cytidine deaminase signatures during indolent chronic lymphocytic leukaemia evolution. Nat Commun.

[9] Palacios F, Moreno P, Morande P (2010). High expression of AID and active class switch recombination might account for a more aggressive disease in unmutated CLL patients: link with an activated microenvironment in CLL disease. Blood.

[10] Robbiani DF, Bothmer A, Callen E (2008). AID is required for the chromosomal breaks in c-myc that lead to c-myc/IgH translocations. Cell.

[11] Bolli N, Maura F, Minvielle S (2018). Genomic patterns of progression in smoldering multiple myeloma. Nat Commun.

[12] Swaminathan S, Klemm L, Park E (2015). Mechanisms of clonal evolution in childhood acute lymphoblastic leukemia. Nat Immunol.

[13] Gruber TA, Chang MS, Sposto R, Muschen M (2010). Activation-induced cytidine deaminase accelerates clonal evolution in BCR-ABL1-driven B-cell lineage acute lymphoblastic leukemia. Cancer Res.

[14] Klemm L, Duy C, Iacobucci I (2009). The B cell mutator AID promotes B lymphoid blast crisis and drug resistance in chronic myeloid leukemia. Cancer Cell.

[15] Rodríguez-Hernández G, Opitz FV, Delgado P (2019). Infectious stimuli promote malignant B-cell acute lymphoblastic leukemia in the absence of AID. Nat Commun.

[16] Tokoyoda K, Zehentmeier S, Hegazy AN (2009). Professional memory CD4+ T lymphocytes preferentially reside and rest in the bone marrow. Immunity.

[17] Traxel S, Schadt L, Eyer T (2019). Bone marrow T helper cells with a Th1 phenotype induce activation and proliferation of leukemic cells in precursor B acute lymphoblastic leukemia patients. Oncogene.

[18] Mordasini V, Ueda S, Aslandogmus R (2017). Activation of ATR-Chk1 pathway facilitates EBV-mediated transformation of primary tonsillar B-cells. Oncotarget.

[19] Perkins JR, Dawes JM, McMahon SB (2012). ReadqPCR and NormqPCR: R packages for the reading, quality checking and normalisation of RT-qPCR quantification cycle (Cq) data. BMC Genomics.

[20] Schreiber E, Matthias P, Müller MM, Schaffner W (1989). Rapid detection of octamer binding proteins with ‘mini extracts’, prepared from a small number of cells. Nucleic Acids Res.

[21] Meyer LH, Eckhoff SM, Queudeville M (2011). Early relapse in ALL is identified by time to leukemia in NOD/SCID mice and is characterized by a gene signature involving survival pathways. Cancer Cell.

[22] Downing JR, Wilson RK, Zhang J (2012). The pediatric cancer genome project. Nat Genet.

[23] Blokzijl F, Janssen R, van Boxtel R, Cuppen E (2018). Mutational patterns: comprehensive genome-wide analysis of mutational processes. Genome Med.

[24] Welcome Sanger Institute COSMIC mutational signatures—version 3. https://cancer.sanger.ac.uk/cosmic/signatures. Accessed 27 Feb 2020

[25] Álvarez-Prado ÁF, Pérez-Durán P, Pérez-García A (2018). A broad atlas of somatic hypermutation allows prediction of activation-induced deaminase targets. J Exp Med.

[26] Obenchain V, Lawrence M, Carey V (2014). VariantAnnotation: a bioconductor package for exploration and annotation of genetic variants. Bioinformatics.

[27] Tate JG, Bamford S, Jubb HC (2019). COSMIC: the catalogue of somatic mutations in cancer. Nucleic Acids Res.

[28] Gu Z, Churchman ML, Roberts KG (2019). PAX5-driven subtypes of B-progenitor acute lymphoblastic leukemia. Nat Genet.

[29] Wang CL, Harper RA, Wabl M (2004). Genome-wide somatic hypermutation. Proc Natl Acad Sci.

[30] Muramatsu M, Sankaranand VS, Anant S (1999). Specific expression of activation-induced cytidine deaminase (AID), a novel member of the RNA-editing deaminase family in germinal center B cells. J Biol Chem.

[31] Kajiwara K, Shinazawa M, Morishima H, Yanagihara Y (2004). Differential effect of IL-4 and IL-13 on the expression of recombination-activating genes in mature B cells from human peripheral blood. Cell Immunol.

[32] Tran TH, Nakata M, Suzuki K (2010). B cell-specific and stimulation-responsive enhancers derepress Aicda by overcoming the effects of silencers. Nat Immunol.

[33] Lin J-X, Migone T-S, Tseng M (1995). The role of shared receptor motifs and common stat proteins in the generation of cytokine pleiotropy and redundancy by IL-2, IL-4, IL-7, IL-13, and IL-15. Immunity.

[34] Ma X, Liu Y, Liu Y (2018). Pan-cancer genome and transcriptome analyses of 1,699 paediatric leukaemias and solid tumours. Nature.

[35] Maura F, Degasperi A, Nadeu F (2019). A practical guide for mutational signature analysis in hematological malignancies. Nat Commun.

[36] Nik-Zainal S, Alexandrov LB, Wedge DC (2012). Mutational processes molding the genomes of 21 breast cancers. Cell.

[37] Feldhahn N, Henke N, Melchior K (2007). Activation-induced cytidine deaminase acts as a mutator in BCR-ABL1-transformed acute lymphoblastic leukemia cells. J Exp Med.

[38] van Zutven LJCM, van Drunen E, de Bont JM (2005). CDKN2 deletions have no prognostic value in childhood precursor-B acute lymphoblastic leukaemia. Leukemia.

[39] Storb U (2014). Why does somatic hypermutation by AID require transcription of its target genes?. Advances in immunology.

[40] Chang MT, Asthana S, Gao SP (2016). Identifying recurrent mutations in cancer reveals widespread lineage diversity and mutational specificity. Nat Biotechnol.

[41] Shi Y, Zhao X, Durkin L (2016). Aberrant activation-induced cytidine deaminase expression in Philadelphia chromosome-positive B-cell acute lymphoblastic leukemia. Hum Pathol.

[42] Högerkorp C-M, Borrebaeck CAK (2006). The human CD77-B cell population represents a heterogeneous subset of cells comprising centroblasts, centrocytes, and plasmablasts, prompting phenotypical revision. J Immunol.

[43] Ranuncolo SM, Polo JM, Dierov J (2007). Bcl-6 mediates the germinal center B cell phenotype and lymphomagenesis through transcriptional repression of the DNA-damage sensor ATR. Nat Immunol.

[44] Phan RT, Dalla-Favera R (2004). The BCL6 proto-oncogene suppresses p53 expression in germinal-centre B cells. Nature.

[45] Han J-H, Akira S, Calame K (2007). Class switch recombination and somatic hypermutation in early mouse B cells are mediated by B cell and toll-like receptors. Immunity.

[46] Os A, Bürgler S, Ribes AP (2013). Chronic lymphocytic leukemia cells are activated and proliferate in response to specific T helper cells. Cell Rep.

[47] Poltoratsky V, Prasad R, Horton JK, Wilson SH (2006). Down-regulation of DNA polymerase beta accompanies somatic hypermutation in human BL2 cell lines. DNA Repair (Amst).

[48] Geng H, Hurtz C, Lenz KB (2015). Self-enforcing feedback activation between BCL6 and pre-B cell receptor signaling defines a distinct subtype of acute lymphoblastic leukemia. Cancer Cell.

