# Supplementary material for: Support of BCP-ALL-cells by autologous bone marrow Th-cells involves induction of AID expression but not widespread AID off-target mutagenesis
Source: Cancer Immunol Immunother. 2021 Jan 28;70(8):2275–89. doi: 10.1007/s00262-020-02835-x (PMC8289808; doi:10.1007/s00262-020-02835-x)
Supplement: Supplementary file 1 — Supplementary file1 (PDF 88 KB) [file 262_2020_2835_MOESM1_ESM.pdf]

Cancer Immunology, Immunotherapy (submitted in 2020) – Sabrina Traxel et al.

Supplementary Figure 1

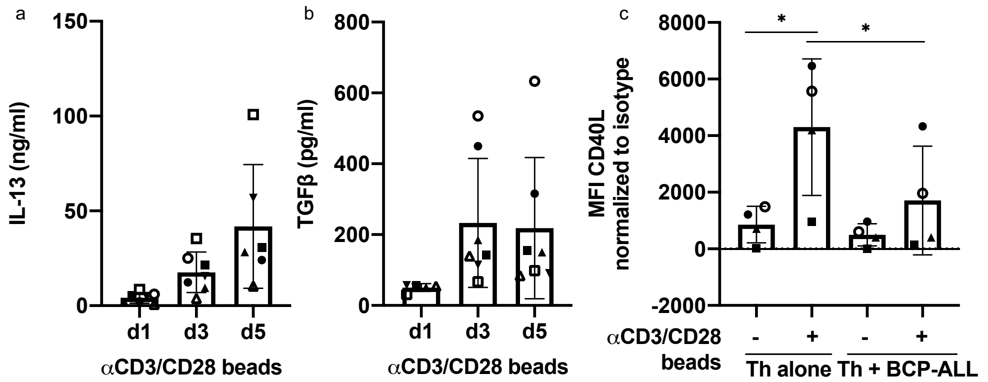

Supplementary Figure 1 IL-13, TGFβ and CD40L are expressed by bone marrow Th cells. a-b) Rested Th cells were stimulated using anti-CD3/CD28 beads. Supernatants were collected after 1-5 days of stimulation. Cytokine concentration was determined by ELISA. c) Th cells were stimulated using anti-CD3/CD28 beads and/or co-cultured with autologous BCP-ALL cells for 3d. CD40L surface expression was analyzed using flow cytometry. P-values were calculated using two-tailed t-tests. P<0.05 *
