# Supplementary material for: Support of BCP-ALL-cells by autologous bone marrow Th-cells involves induction of AID expression but not widespread AID off-target mutagenesis
Source: Cancer Immunol Immunother. 2021 Jan 28;70(8):2275–89. doi: 10.1007/s00262-020-02835-x (PMC8289808; doi:10.1007/s00262-020-02835-x)
Supplement: Supplementary file 2 — Supplementary file2 (PDF 307 KB) [file 262_2020_2835_MOESM2_ESM.pdf]

Cancer Immunology, Immunotherapy (submitted in 2020) – Sabrina Traxel et al.

Supplementary Figure 2

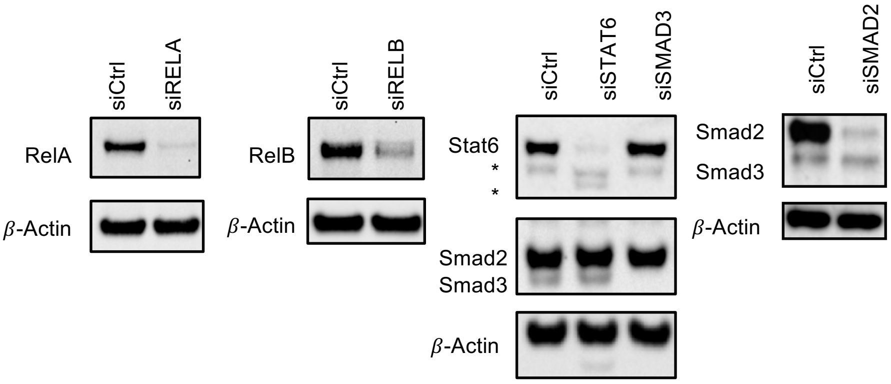

Supplementary Figure 2 Confirmation of siRNA induced knock-down. RS4;11 were electroporated with siRNA's targeting different transcription factors or non-targeting siRNA (siCtrl). Total protein was extracted 72h (RelA, RelB, Stat6, Smad3) or 48h (Smad2) after transfection and analyzed by Western Blotting. β-Actin was used as loading control. *unspecific bands.
